# Exercise Reduces Liver Lipids and Visceral Adiposity in Patients With Nonalcoholic Steatohepatitis in a Randomized Controlled Trial

**DOI:** 10.1016/j.cgh.2016.07.031

**Published:** 2017-01

**Authors:** David Houghton, Christian Thoma, Kate Hallsworth, Sophie Cassidy, Timothy Hardy, Alastair D. Burt, Dina Tiniakos, Kieren G. Hollingsworth, Roy Taylor, Christopher P. Day, Stuart McPherson, Quentin M. Anstee, Michael I. Trenell

**Affiliations:** ∗ Institute of Cellular Medicine, Newcastle University, Newcastle upon Tyne, United Kingdom; ‡ Liver Unit, Newcastle Upon Tyne Hospitals NHS Trust, Freeman Hospital, Newcastle Upon Tyne, United Kingdom; § Faculty of Health Sciences, The University of Adelaide, Adelaide, SA, Australia

**Keywords:** NAFLD, Inflammation, Therapy, Body Composition, ALT, alanine aminotransferase, AST, aspartate aminotransferase, BMI, body mass index, CK-18, cytokeratin-18, hsCRP, high sensitivity C-reactive protein, IL, interleukin, NAFLD, nonalcoholic fatty liver disease, NASH, nonalcoholic steatohepatitis, HTGC, hepatic triglyceride content, TNF-α, tumor necrosis factor α

## Abstract

**Background & Aims:**

Pharmacologic treatments for nonalcoholic steatohepatitis (NASH) are limited. Lifestyle interventions are believed to be effective in reducing features of NASH, although the effect of regular exercise, independent of dietary change, is unclear. We performed a randomized controlled trial to study the effect of exercise on hepatic triglyceride content (HTGC) and biomarkers of fibrosis in patients with NASH.

**Methods:**

Twenty-four patients (mean age, 52 ± 14 y; body mass index, 33 ± 6 kg/m^2^) with sedentary lifestyles (<60 min/wk of moderate–vigorous activity) and biopsy-proven NASH were assigned randomly to groups that exercised (n = 12) or continued standard care (controls, n = 12) for 12 weeks while maintaining their weight. The exercise (cycling and resistance training) was supervised at an accredited sports center and supervised by a certified exercise specialist and recorded 3 times per week on nonconsecutive days. We measured HTGC, body composition, circulating markers of inflammation, fibrosis, and glucose tolerance at baseline and at 12 weeks.

**Results:**

Compared with baseline, exercise significantly reduced HTGC (reduction of 16% ± 24% vs an increase of 9% ± 15% for controls; *P* < .05), visceral fat (reduction of 22 ± 33 cm^2^ vs an increase of 14 ± 48 cm^2^ for controls; *P* < .05), plasma triglycerides (reduction of 0.5 ± 1.0 mmol/L vs an increase of 0.3 ± 0.4 mmol/L for controls; *P* < .05), and γ-glutamyltransferase (reduction of 10 ± 28 U/L^–^^1^ vs a reduction of 17 ± 38 U/L^−1^ for controls; *P* < .05). There were no effects of exercise on liver enzyme levels, metabolic parameters, circulatory markers of inflammation (levels of interleukin 6, tumor necrosis factor-α, or C-reactive protein) and fibrosis.

**Conclusions:**

In a randomized controlled trial, 12 weeks of exercise significantly reduced HTGC, visceral fat, and plasma triglyceride levels in patients with NASH, but did not affect circulating markers of inflammation or fibrosis. Exercise without weight loss therefore affects some but not all factors associated with NASH. Clinical care teams should consider exercise as part of a management strategy of NASH, but weight management strategies should be included. Larger and longer-term studies are required to determine the effects of exercise in patients with NASH. ISRCTN registry.com: ISRCTN16070927.

Nonalcoholic fatty liver disease (NAFLD) encompasses a spectrum of liver conditions ranging from simple steatosis through nonalcoholic steatohepatitis (NASH), fibrosis, and cirrhosis.^1^ Current evidence from a large single-center serial biopsy study, supported by a recent meta-analysis, indicates that approximately 40% of NAFLD cases will show progressive fibrosis during a median 6.6-year follow-up evaluation.^2^, ^3^ Although the accumulation of various lipids in the liver is the prerequisite for NAFLD, recent evidence suggests that inflammatory biomarkers also could play a role in the development of NASH.^4^

Currently, there are no approved pharmacologic therapies for managing NAFLD, although a number of promising agents are in trial.^5^ Lifestyle interventions, incorporating weight loss and increased physical activity/exercise, remain the cornerstone of NAFLD management,^6^, ^7^, ^8^, ^9^ however, implementation remains difficult.^10^ Evidence supporting the effect of physical activity independent of weight loss is limited, impeding successful translation into clinical practice. Increasing physical activity through structured exercise has shown a significant beneficial effect on hepatic triglyceride content (HTGC).^7^, ^11^, ^12^ To date, only 1 study has assessed the effect of exercise in biopsy-proven NASH and reported no change in HTGC or fibrosis.^9^ However, the study did not include a control group and used the percentage of hepatocytes affected to assess HTGC, limiting sensitivity.

The primary objective of this randomized controlled trial was to determine the effects of exercise, without weight loss, on HTGC in adults with biopsy-confirmed NASH. The secondary aims were to determine the effect of exercise on mediators of NASH: abdominal adiposity, glucose control, circulating markers of inflammation, and noninvasive markers of fibrosis.

## Materials and Methods

**Supplementary Figure 1:**
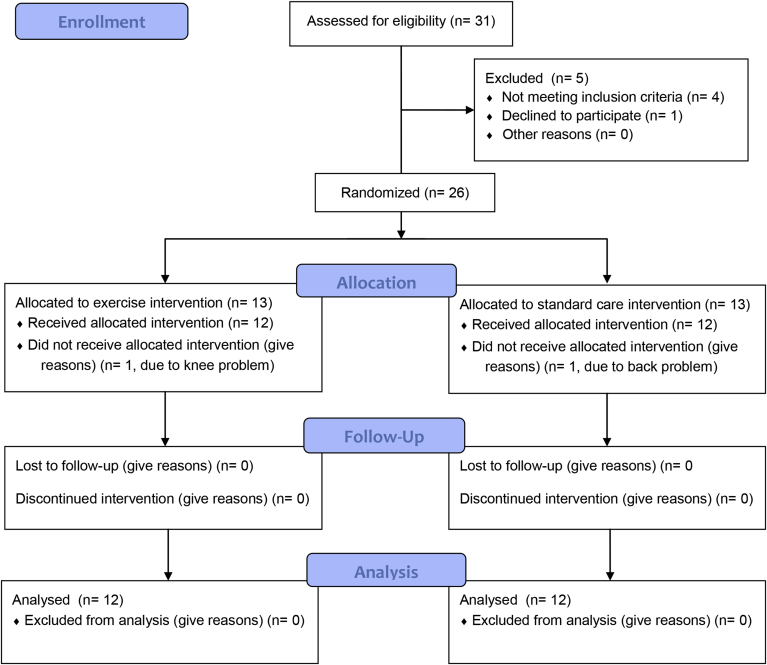
CONSORT Flow Diagram.

Thirty-one patients with histologically characterized NASH (age, 59 ± 12 y; body mass index [BMI], 35 ± 5 kg/m^2^) were screened for study entry. Patients with evidence of other liver disease or a history of excessive alcohol consumption (alcohol intake >20 g/d for women or >30 g/d for men) were excluded (Table 1). The study protocol was approved by the Sunderland Research Ethics Committee, UK, and all patients provided written informed consent. Liver biopsy specimens were scored histologically by 2 expert histopathologists (A.D.B. and D.T.) according to the NASH Clinical Research Network criteria.^13^ The NAFLD activity score was graded between 0 and 8, and fibrosis was staged from 0 to 4 as previously performed^14^ (Table 2). Other exclusion criteria included the following: heart or kidney disease, implanted ferrous metal, pre-existing medical conditions preventing participation in the exercise program, insulin-sensitizing treatment, or dietary change over the preceding 6 months. Five patients were excluded during screening because of abnormal electrocardiographs and 1 patient withdrew because of claustrophobia during magnetic resonance imaging scanning (see Consolidated Standards of Reporting Trials diagram, Supplementary Figure 1).

Twenty-six sedentary (<60 min/wk of moderate-vigorous activity) patients were assigned randomly using a permuted blocks method to either exercise (n = 13) or standard care (n = 13) (Supplementary Figure 1). Participant characteristics are summarized in Table 1. After an initial screening visit, patients underwent a full medical history, physical examination, and progressive exercise test to screen for any undiagnosed cardiac disease as previously described.^12^ HTGC, body composition, fasted blood samples, including inflammatory (interleukin 6 [IL6], tumor necrosis factor α [TNF-α], and high-sensitivity C-reactive protein [hsCRP] (V-PLEX K15049D plate; Meso-Scale, Rockville, MD; and Roche Diagnostics Ltd, Burgess Hill, UK, respectively) and fibrosis markers (cytokeratin-18 [CK-18] using the M30-Apoptosense emzyme-linked immunosorbent assay kit; PEVIVA, Bromma, Sweden),^15^ and a 2-hour frequently sampled oral glucose tolerance test were measured at baseline and at 12 weeks.^12^, ^16^, ^17^

### Study Intervention

Exercise was supervised by an accredited exercise specialist and recorded to ensure adherence 3 times per week on nonconsecutive days for 12 weeks.^18^, ^19^ The exercise program consisted of aerobic (cycling) and resistance training, and is detailed in Supplementary Methods. All patients were instructed not to alter their diet and to maintain their current weight throughout the study.

### Statistics

Sample size was calculated based on change in HTGC from previous data in NAFLD^12^; an 80% power of detecting a 10% relative difference between group change in liver HTGC with a SD of 9.0 and a 1-sided 0.05 significance required n = 11 per group. We recruited 13 per sample to allow 2 drop-outs per group. Normality was assessed using a Kolmogorov–Smirnov test and logarithmically transformed if not normally distributed. Between-group differences were evaluated using an unpaired t test and within-group differences using a paired t test (2 way). Treatment × time interactions were assessed using a 2-way analysis of variance. Analyses of covariance were used to test for between-group differences in outcome variables while controlling for baseline values. Bivariate correlations using Pearson rank correlations were conducted to investigate any associations between HTGC, body composition, triglyceride levels, glucose control, biomarkers of inflammation, and NAFLD fibrosis marking systems. Statistical significance was set at a P value of less than .05. Statistical analyses were performed using SPSS statistical analysis software (version 19; IBM, Chicago, Illinois). All authors had access to the study data and reviewed and approved the final manuscript.

## Results

Twenty-six patients were randomized, with 2 patients withdrawing owing to pre-existing knee and back problems (Supplementary Figure 1). Twenty-four patients completed the study, with all subjects in the exercise group completing the 36-session exercise program. The control and exercise groups were well matched (Table 1), with no significant differences in age (51 ± 16 vs 54 ± 12 y) or BMI (33 ± 5 vs 33 ± 7 kg/m^2^). Baseline histologic scores and fibrosis staging are shown in Table 2.

### Body Mass Index, Body Composition, and Hepatic Triglyceride Content

**Figure 1:**
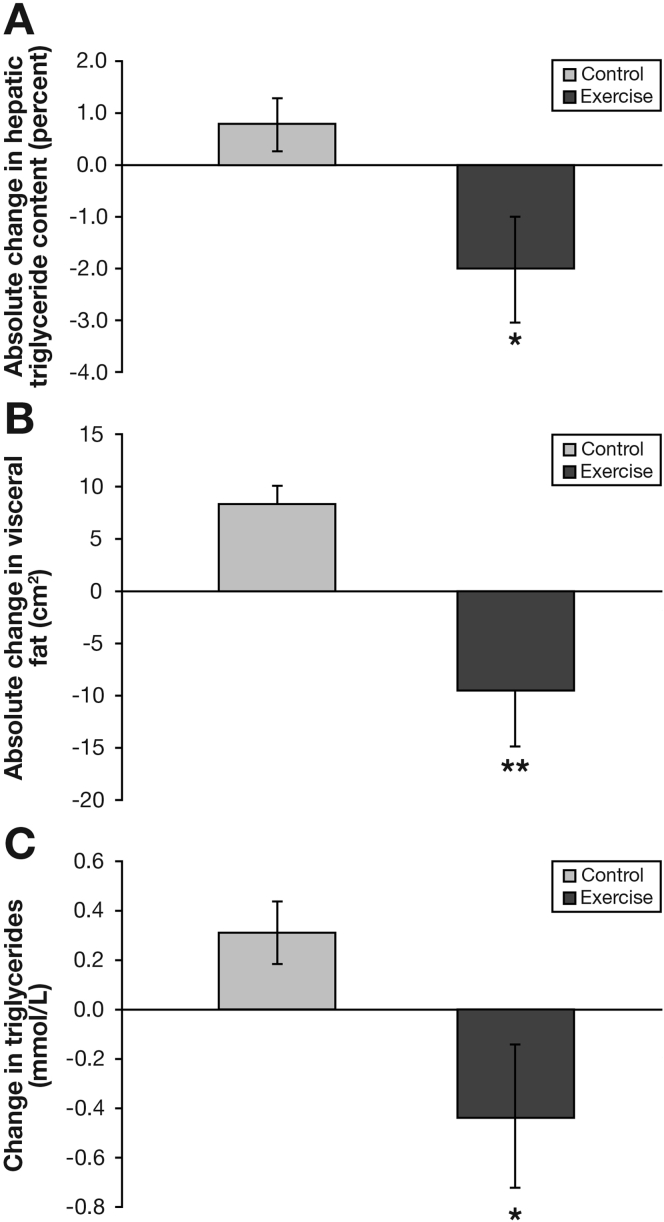
Effect of 12 weeks exercise training (Exercise) or standard care (Control) on absolute changes in intrahepatic lipid (*A*), visceral fat (*B*), and triglycerides (*C*) from baseline. Values are means ± SE.M (n = 24) ^∗^, significantly different from control (P < .05). ^∗∗^, significantly different from control (P < .01).

During the study, BMI, weight, and subcutaneous fat remained constant in both groups (Tables 1 and 3). Visceral fat decreased by 12% in the exercise group and increased by 7% in the control group (*P* < .01) (Figure 1 and Table 3). Exercise increased lean body mass by 4% vs 0% in the control group (*P* < .05), and reduced fat mass by 6% vs 0% in the control group, although the latter was not statistically significant. Exercise produced a 16% reduction in HTGC compared with an 8% increase in the control group (*P* < .05) (Figure 1 and Table 3).

### Blood Lipid and Liver Enzyme Levels

There was a time by treatment interaction for triglyceride levels, with exercise eliciting a reduction of 23% vs a 13% increase in the control group (*P* < .05). There was also a significant reduction of 13% in γ-glutamyltransferase after exercise (*P* < .05). No other significant changes in blood biochemistry were observed in either group (Table 1).

### Metabolic Control

The exercise group showed a -17% vs -7% reduction in fasting insulin level, and decreased homeostatic model assessment of insulin resistance of -17% vs +6%, although these values did not reach statistical significance (Table 3). There were no differences in any other glucose control variables after the intervention in either group or between groups (Table 3). No time by treatment interaction for fasting blood glucose, fasting insulin, glycated hemoglobin, homeostatic model assessment of insulin resistance, or glucose area under the curve for the frequently sampled oral glucose tolerance test were observed (Table 3).

### Circulatory Inflammation and Noninvasive Fibrosis Markers

There was no significant time by treatment interaction for exercise on circulating inflammatory biomarkers, CK-18, hsCRP, or the noninvasive markers of fibrosis (NAFLD fibrosis score, aspartate aminotransferase [AST]/alanine aminotransferase [ALT] ratio, and enhanced liver fibrosis test) (Table 4).

### Correlations

HTGC was associated positively with visceral fat at baseline and after the intervention (r = 0.49, *P* = .01; and r = 0.36, *P* = .04, respectively). There was also a positive correlation between the changes in HTGC and visceral fat after the intervention (r = 0.39, *P* = .03). There were no other significant correlations between HTGC, body composition, glucose control, biomarkers of inflammation, or fibrosis markers at baseline or postintervention.

## Discussion

This RCT examined the effects of exercise, independent of weight loss, in adults with histologically confirmed NASH. The data show that 12 weeks of exercise resulted in the following: (1) a 16% reduction in liver fat, (2) a 12% reduction in visceral fat, (3) a 23% reduction in circulating triglyceride levels, and (4) a 4% increase in lean body mass. However, 12 weeks of exercise had no significant effect on glucose control, circulating markers of inflammation, liver enzyme levels, or NAFLD activity score.

Lifestyle modification combining dietary change and exercise produces a robust reduction in HTGC and even reversal of inflammation with more than 10% weight loss in people with NASH,^6^, ^8^ data on exercise without weight loss are lacking. We show that 12 weeks of exercise therapy resulted in a 16% reduction in HTGC, independent of weight loss in people with NASH. The changes in HTGC reported here are in line with previous reports of exercise without weight loss in people with NAFLD^11^, ^12^ and show the effectiveness of exercise in reducing HTGC in NASH. Although consistent with earlier reports in NAFLD, our observation contrasts with the only other study looking specifically at exercise in NASH, which reported an apparent stability of HTGC after 6 months of exercise.^9^ The difference between these studies most likely can be explained by the greater sensitivity of proton magnetic resonance spectroscopy (as used in this study) vs histologic assessment of the percentage of hepatocytes affected in the small number of cases in the previous study and the well-controlled design of the present study. Our HTGC data suggest that exercise may hold therapeutic benefits for people with NASH.

Although the reduction in HTGC with exercise is encouraging, the changes reported here should be placed in context of studies using a combination of weight loss and exercise in NAFLD, which see a relative reduction of between 42% and 81% in HTGC after a mean weight reduction of 4% to 14%.^7^ Weight loss and exercise yielding a 10% reduction in body weight can reverse NASH,^6^, ^8^ highlighting the summative benefits of weight loss and exercise. Although weight loss undoubtedly is effective in reducing HTGC, the difficulty of maintaining weight loss in the long term is well documented.^10^ As such, the optimal clinical value of exercise upon liver health for people with NASH appears likely to be an adjunct to caloric restriction. However, exercise without weight loss may represent an alternative therapy for patients who find weight loss difficult.

The mechanisms underlying the change in HTGC after exercise in NASH reflect changes in energy balance, circulatory lipids, and insulin sensitivity. Without any change in body weight, exercise reduced visceral fat by 12%. To date, only 1 study has assessed the effects of exercise in biopsy-proven NASH,^9^ however, the current RCT assessed the effects of exercise on HTGC, metabolic control, body composition, alongside inflammatory and fibrosis markers in patients with NASH. Visceral fat is reported to be linked directly with liver inflammation and fibrosis, independent of insulin resistance and hepatic steatosis.^20^ The precise mechanism of how visceral fat applies its detrimental effects on liver metabolism and fibrotic and inflammatory consequences remains unclear, although an influx of fatty acids and synthesis of cytokines and adipokines has been shown to promote liver lipid accumulation, insulin resistance, and inflammation.^20^, ^21^ The present data support the close relationship between visceral fat and HTGC, with baseline visceral fat correlating with HTGC and the change in visceral fat with exercise correlating with the change in HTGC.

The apparent stability in metabolic control here is surprising given the reduction in HTGC and visceral fat, and the close link between HTGC, hepatic insulin sensitivity, and endogenous glucose production.^22^, ^23^ Despite a 16% reduction in HTGC, 19 patients remained above the clinical 5% HTGC diagnosis, suggesting that a larger reduction in HTGC is required to improve hepatic insulin sensitivity and glucose control significantly, as recently shown.^24^, ^25^ Irrespective of the effects of exercise on glycemic control, the selective reduction of both visceral fat and HTGC with exercise, independent of weight loss, supports exercise as an adjunct therapy for NASH, as previously shown in NAFLD.^11^, ^12^

In this study there was a reduction in CK-18, a marker of apoptosis and a defining pathologic feature of NASH, known to correlate with liver damage and fibrosis.^15^ However, TNF-α, IL6, and hsCRP all remained stable in the exercise group, despite changes in HTGC and visceral fat. Although increased liver lipid is a prerequisite of NASH,^26^ dysregulated cytokine metabolism plays an important role in disease progression.^4^ Exercise studies in coronary artery disease, type 2 diabetes mellitus, and NAFLD have reported reductions in circulating cytokine levels (IL6 and TNF-α)^27^, ^28^ and CK-18,^29^ raising the possibility of a protective effect of exercise. However, a combined weight loss and exercise program in people with NASH also showed a stability in circulatory cytokine levels, with the exception of IL6, which was reduced.^30^ Importantly, circulatory inflammatory markers do not represent inflammation inside of the liver. Because hepatic inflammatory markers were not assessed directly, HTGC may represent a better biomarker of free fatty acid flux, oxidative stress, and ER stress that result in steatosis and progressive liver damage.^31^ Although there are clear biological links between inflammation and NASH, further studies are required to understand how lifestyle management may be optimized to address these.

Liver enzyme levels (ALT, AST) and noninvasive scores of liver disease (ALT/AST ratio, NAFLD fibrosis score, and an increased liver fibrosis test) did not change with 12 weeks of exercise therapy. Furthermore, changes in HTGC were not correlated with liver enzyme levels or noninvasive scores of liver disease. Our study used liver enzyme levels and noninvasive measures of liver disease because follow-up biopsies to assess liver histology were not ethically permitted. However, biopsy data show that significant weight loss as a result of calorie restriction and exercise improves liver histology.^6^, ^8^ The lack of change in liver enzyme levels and biomarkers of liver disease after exercise suggest that exercise without weight loss may be insufficient to influence liver fibrosis directly. However, without a follow-up biopsy it is difficult to truly ascertain the impact of exercise alone on NASH. The present data support longer and larger studies investigating the differential and combined effects of exercise and weight loss in NASH to confirm their effects on disease progression.

### Limitations

The present study was not without limitation. Liver fibrosis was not measured directly from biopsy after exercise. However, because recent work has suggested that steatosis and NASH may be more interchangeable than previously thought^2^ it was ethically difficult to justify exposing volunteers to the risk of a repeat biopsy within such a short period of time. Although powered sufficiently for the primary outcome, the sample size may be insufficient to define secondary outcomes. The length of the study, although comparable with previous exercise studies in NAFLD, may not have been long enough in duration to observe any improvements in histology and circulating inflammation in NASH.

## Conclusions

Exercise was well tolerated and reduced HTGC, visceral fat, and circulating triglycerides in adults with biopsy-proven NASH, independent of weight loss, but without any apparent impact on circulating markers of inflammation or noninvasive scores of liver disease. These data suggest that increased exercise in the absence of weight loss is effective at reducing HTGC, but may be less efficacious at ameliorating steatohepatitis over a 12-week program. Clinically, exercise has a significant beneficial effect on HTGC and visceral fat. However, the optimal clinical value of exercise for people with NASH appears likely to be an adjunct to caloric restriction.

## Figures and Tables

**Table 1:** Subject Characteristics

	Control (n = 12)			Exercise (n = 12)			Time × treatment interaction
	Baseline	Post-treatment	*P* value	Baseline	Post-treatment	*P* value	*P* value
Anthropometry							
Age, *y*	51 (16)	-		54 (12)	-		
BMI, *kg/m*^*2*^	33 (5)	34 (5)	.18	33 (7)	33 (7)	.12	.77
Weight, *kg*	94 (9)	95 (9)	.15	90 (18)	91 (18)	.12	.86
Vo_2peak_, *mL/kg*^*-1*^*/min*^*-1*^	21 (5)		-	25 (8)			
Metabolic							
ALT level, *U/L*^*-1*^	81 (59)	75 (52)	.28	53 (25)	52 (18)	.31	.44
AST level, *U/L*^*-1*^	59 (27)	58 (30)	.33	41 (14)	45 (12)	.17	.30
GGT level, *U/L*^*-1*^	113 (78)	96 (53)	.08	66 (46)	56 (33)	.05^a^	.82
Total cholesterol level, *mmol/L*	4.9 (1.3)	5.2 (1.3)	.07	4.7 (1.4)	4.5 (1.3)	.26	.20
Triglyceride level, *mmol/L*	2.0 (0.9)	2.3 (1.0)	.02^a^	2.2 (1.0)	1.7 (0.8)	.09	.03^b^
Platelets, *×10 g/L*	261 (83)	258 (75)	.42	208 (42)	214 (44)	.06	.35
Albumin level, *g/L*	46.5 (2.5)	46.7 (3.3)	.35	45.4 (3.8)	45.7 (3.4)	.31	.93

NOTE. Values are means (SD).

GGT, γ-glutamyltransferase; VO_2_peak, aerobic capacity.

a Significant difference in baseline vs post-intervention (*P* < .05).

b Significant difference in time × treatment interaction (*P* < .05).

**Table 2:** Baseline Liver Histology and NAFLD Fibrosis Score

	Control (n = 12)	Exercise (n = 12)	*P* value
NAS	5 (2–7)	5 (3–7)	.61
Steatosis	1 (1–3)	2 (1–3)	.80
Inflammation	1 (0–2)	2 (1–2)	.47
Ballooning	1 (0–2)	1 (1–2)	.52
Fibrosis stage	3 (0–3)	3 (2–3)	.28
0	1 (4%)	0 (0%)	
1	2 (8%)	0 (0%)	
2	3 (13%)	5 (21%)	
3	6 (25%)	7 (29%)	
4	0 (0%)	0 (0%)	

NOTE. Baseline liver histology and fibrosis stage are shown as a median (range) for all patients.

NAS, NAFLD activity score.

**Table 3:** Hepatic Triglyceride Content, Adipose Tissue, Body Composition, and Metabolic Control

	Control (n = 12)			Exercise (n = 12)			Time × treatment interaction
	Baseline	Post-treatment	*P* value	Baseline	Post-treatment	*P* value	*P* value
Hepatic triglyceride content, *%*	10 (5)	11 (5)	.08	12 (9)	10 (6)	.04^a^	.02^b^
Visceral adipose tissue, *cm*^*2*^	173 (75)	187 (53)	.02^a^	191 (86)	169 (50)	.04^a^	.01^c^
Subcutaneous adipose tissue, *cm*^*2*^	396 (124)	337 (181)	.24	409 (113)	318 (158)	.08	.07
Fat mass, *kg*	38 (9)	38 (8)	.37	35 (15)	33 (15)	.07	.24
Lean body mass, *kg*	57 (7)	57 (7)	.10	56 (10)	58 (10)	.01^d^	.51
Fasting glucose, *mmol/L*	5.8 (1.5)	5.8 (1.8)	.41	6.7 (1.7)	6.6 (1.6)	.38	.80
Fasting insulin, *pmol/L*	98 (58)	91 (52)	.18	118 (75)	99 (44)	.23	.96
HOMA-IR	1.6 (1.1)	1.7 (1.0)	.18	2.3 (1.4)	1.9 (0.8)	.26	.53
HbA_1c_, *mmol/mol*	47 (11)	49 (15)	.16	52 (14)	50 (13)	.14	.13
fsOGTT, AUC	838 (191)	879 (270)	.23	1016 (279)	980 (329)	.20	.91

NOTE. Values are means (± SD).

AUC, area under the curve; fsOGTT, frequently sampled oral glucose tolerance test; HbA1c, glycated hemoglobin; HOMA-IR, homeostasis model of insulin resistance.

a Significant difference in baseline vs postintervention (*P* < .05).

b Significant difference in time × treatment interaction (*P* < .05).

c Significant difference in time × treatment interaction (*P* < .01).

d Significant difference in baseline vs postintervention (*P* < .01).

**Table 4:** Liver Enzyme Levels, NAFLD Activity Score, Increased Liver Fibrosis Test, and Circulatory Cytokines/Inflammation

	Control(n = 12)			Exercise(n = 12)			Time × treatment interaction
	Baseline	Post-treatment	*P* value	Baseline	Post-treatment	*P* value	*P* value
ALT/AST ratio	0.92 (0.44)	0.93 (0.36)	.42	0.86 (0.32)	0.92 (0.36)	.10	.63
NAFLD fibrosis score	−0.95 (1.43)	−0.98 (1.53)	.36	−1.51 (1.00)	−1.50 (1.12)	.47	.80
Increased liver fibrosis test	8.8 (0.9)	8.8 (1.1)	.46	9.4 (1.1)	9.5 (1.2)	.35	.76
*TNF-α*, pg/mL	2.3 (0.7)	2.3 (0.7)	.25	2.2 (0.6)	2.4 (0.7)	.17	.25
*IL6*, pg/mL	2.4 (4.2)	2.0 (2.7)	.38	1.4 (0.7)	1.7 (1.7)	.44	.77
hsCRP, mg/L	2.1 (1.5)	3.1 (3.0)	.48	2.8 (2.4)	4.9 (4.9)	.21	.71
CK-18, U/L	842 (1140)	781 (1013)	.44	627 (764)	441 (275)	.43	.82

NOTE. Values are means (± SD).

## Supplementary Methods

### Protocol

Twenty-six sedentary (<60 min/wk of moderate-vigorous activity) patients were assigned randomly using a permuted blocks method with an independent member of the research group^1^ to either exercise intervention (n = 13) or standard care (n = 13) (Supplementary Figure 1). Participant characteristics are summarized in Table 1. After an initial screening visit, patients underwent a full medical history, physical examination, and progressive exercise test to screen for any undiagnosed cardiac disease as previously described.^12^ Body composition, HTGC and abdominal fat were measured using air-displacement plethysmography and a 3.0 Tesla Philips Achieva magnetic resonance image scanner (Guilford, Surrey), respectively, also as previously described.^2^, ^3^, ^4^, ^5^, ^6^

### Blood Analyses

A 75-g glucose load (Lucozade Original; Glaxo SmithKline, Brentford, UK) was then consumed within 5 minutes and blood samples then were taken at 15, 30, 45, 60, 75, 90, and 120 minutes. Samples were analyzed for whole-blood glucose (YSI 2300 Stat Plus-D; Yellow Springs Instruments, Yellow Springs, OH), and plasma insulin (Coat-A-Count Insulin RIA kit; Diagnostic Products Corporation, CA). The area under the curve for the resulting glucose response profile was calculated using the trapezoidal rule^7^ and insulin resistance was determined using the homeostasis model of insulin resistance.^8^ Fasting samples also were analyzed in a Clinical Pathology Accredited laboratory (Newcastle Upon Tyne Hospital NHS Foundation Trust, Department of Clinical Biochemistry) for the following: ALT, AST, γ-glutamyltransferase, total cholesterol, triglycerides, and glycated hemoglobin. Total cholesterol, triglycerides, ALT, AST, and γ-glutamyltransferase were measured using a Roche Modular P test kit, CK-18 using the M30-Apoptosense emzyme-linked immunosorbent assay kit (PEVIVA),^9^ hsCRP (Roche Diagnostics Ltd, Burgess Hill, UK), and glycated hemoglobin were measured using a TOSOH HLC-723G7 (Tosoh Corporation, Tokyo, Japan). Plasma samples were analyzed for circulatory inflammatory markers using a V-PLEX K15049D plate (Meso-Scale), and serum samples were analyzed for levels of tissue inhibitor of matrix metalloproteinase 1, hyaluronic acid, and aminoterminal peptide of procollagen III at an independent Clinical Pathology Accredited laboratory (North Middlesex University Hospital NHS Foundation Trust).

### Exercise Intervention

Exercise was supervised and performed 3 times per week on nonconsecutive days for 12 weeks, a program we have used successfully for patients with NAFLD^10^ and type 2 diabetes.^11^ The exercise program consisted of aerobic (cycling) and resistance training and is detailed in the Supplementary Clinical Trial Study Protocol. The cycling included a 5-minute warm-up and 3 intervals on a fixed bike for 2 minutes with a 1-minute rest in-between. Exercise intensity was based on the Borg (6–20 points) rating of perceived exertion with bike intervals corresponding to a rating of perceived exertion of 16 to 18 (very hard).^12^ This was followed by a resistance exercise circuit that comprised 5 exercises: hip and knee extension, horizontal row, chest press, vertical row, and knee extension (Precor, Woodinville, WA). Patients were provided with a suitable weight for each resistance exercise based on a rating of perceived exertion of 14 to 16 (hard). The rating of perceived exertion was used to guide intensity for safety, time effectiveness, its translational use in clinical practice, and its effectiveness at determining one repetition maximum.^13^, ^14^ Each session lasted between 45 and 60 minutes. All sessions were conducted at an accredited sports center and supervised by a certified exercise specialist, who recorded progress to ensure adherence and encouraged exercise progression through adding resistance on the bike and increasing the weights lifted as able. This also helped to improve safety, adherence, and the opportunity to resolve any problems. Standard care consisted of volunteers continuing any prescription medication and going for regular monitoring of their condition(s) with their normal general practitioner and/or consultant(s). After 12 weeks of exercise all follow-up tests were performed within 24 hours of the final exercise session.

## References

[1] Anstee Q.M., Targher G., Day C.P. (2013). Progression of NAFLD to diabetes mellitus, cardiovascular disease or cirrhosis. Nat Rev Gastroenterol Hepatol.

[2] McPherson S., Hardy T., Henderson E. (2015). Evidence of NAFLD progression from steatosis to fibrosing-steatohepatitis using paired biopsies: implications for prognosis and clinical management. J Hepatol.

[3] Singh S., Allen A.M., Wang Z. (2015). Fibrosis progression in nonalcoholic fatty liver vs nonalcoholic steatohepatitis: a systematic review and meta-analysis of paired-biopsy studies. Clin Gastroenterol Hepatol.

[4] Park E.J., Lee J.H., Yu G.Y. (2010). Dietary and genetic obesity promote liver inflammation and tumorigenesis by enhancing IL-6 and TNF expression. Cell.

[5] Hardy T., Anstee Q.M., Day C.P. (2015). Nonalcoholic fatty liver disease: new treatments. Curr Opin Gastroenterol.

[6] Vilar-Gomez E., Martinez-Perez Y., Calzadilla-Bertot L. (2015). Weight loss via lifestyle modification significantly reduces features of nonalcoholic steatohepatitis. Gastroenterology.

[7] Thoma C., Day C.P., Trenell M.I. (2012). Lifestyle interventions for the treatment of non-alcoholic fatty liver disease in adults: a systematic review. J Hepatol.

[8] Promrat K., Kleiner D.E., Niemeier H. (2010). Randomized controlled trial testing the effects of weight loss on nonalcoholic steatohepatitis. Hepatology.

[9] Hickman I.J., Byrne N.M., Croci I. (2013). Randomised study of the metabolic and histological effects of exercise in non alcoholic steatohepatitis. J Diabet Metab.

[10] Dudekula A., Rachakonda V., Shaik B. (2014). Weight loss in nonalcoholic fatty liver disease patients in an ambulatory care setting is largely unsuccessful but correlates with frequency of clinic visits. PLoS One.

[11] Johnson N.A., Sachinwalla T., Walton D.W. (2009). Aerobic exercise training reduces hepatic and visceral lipids in obese individuals without weight loss. Hepatology.

[12] Hallsworth K., Fattakhova G., Hollingsworth K.G. (2011). Resistance exercise reduces liver fat and its mediators in non-alcoholic fatty liver disease independent of weight loss. Gut.

[13] Kleiner D.E., Brunt E.M., Van Natta M. (2005). Design and validation of a histological scoring system for nonalcoholic fatty liver disease. Hepatology.

[14] McPherson S., Stewart S.F., Henderson E. (2010). Simple non-invasive fibrosis scoring systems can reliably exclude advanced fibrosis in patients with non-alcoholic fatty liver disease. Gut.

[15] Feldstein A.E., Wieckowska A., Lopez A.R. (2009). Cytokeratin-18 fragment levels as noninvasive biomarkers for nonalcoholic steatohepatitis: a multicenter validation study. Hepatology.

[16] Biaggi R.R., Vollman M.W., Nies M.A. (1999). Comparison of air-displacement plethysmography with hydrostatic weighing and bioelectrical impedance analysis for the assessment of body composition in healthy adults. Am J Clin Nutr.

[17] Longo R., Pollesello P., Ricci C. (1995). Proton MR spectroscopy in quantitative in vivo determination of fat content in human liver steatosis. J Magn Reson Imaging.

[18] Hallsworth K., Thoma C., Hollingsworth K.G. (2015). Modified high-intensity interval training reduces liver fat and improves cardiac function in non-alcoholic fatty liver disease: a randomised controlled trial. Clin Sci (Lond).

[19] Cassidy S., Thoma C., Hallsworth K. (2016). High intensity intermittent exercise improves cardiac structure and function and reduces liver fat in patients with type 2 diabetes: a randomised controlled trial. Diabetologia.

[20] Van der Poorten D., Milner K.L., Hui J. (2008). Visceral fat: a key mediator of steatohepatitis in metabolic liver disease. Hepatology.

[21] Bergman R.N., Kim S.P., Catalano K.J. (2006). Why visceral fat is bad: mechanisms of the metabolic syndrome. Obesity (Silver Spring).

[22] Samuel V.T., Liu Z.X., Qu X. (2004). Mechanism of hepatic insulin resistance in non-alcoholic fatty liver disease. J Biol Chem.

[23] Seppala-Lindroos A., Vehkavaara S., Hakkinen A.M. (2002). Fat accumulation in the liver is associated with defects in insulin suppression of glucose production and serum free fatty acids independent of obesity in normal men. J Clin Endocrinol Metab.

[24] Cuthbertson D.J., Shojaee-Moradie F., Sprung V.S. (2016). Dissociation between exercise-induced reduction in liver fat and changes in hepatic and peripheral glucose homoeostasis in obese patients with non-alcoholic fatty liver disease. Clin Sci (Lond).

[25] Lim E.L., Hollingsworth K.G., Aribisala B.S. (2011). Reversal of type 2 diabetes: normalisation of beta cell function in association with decreased pancreas and liver triacylglycerol. Diabetologia.

[26] Day C.P., James O.F. (1998). Steatohepatitis: a tale of two “hits”?. Gastroenterology.

[27] Goldhammer E., Tanchilevitch A., Maor I. (2005). Exercise training modulates cytokines activity in coronary heart disease patients. Int J Cardiol.

[28] Kadoglou N.P., Perrea D., Iliadis F. (2007). Exercise reduces resistin and inflammatory cytokines in patients with type 2 diabetes. Diabetes Care.

[29] Fealy C.E., Haus J.M., Solomon T.P. (2012). Short-term exercise reduces markers of hepatocyte apoptosis in nonalcoholic fatty liver disease. J Appl Physiol.

[30] Kugelmas M., Hill D.B., Vivian B. (2003). Cytokines and NASH: a pilot study of the effects of lifestyle modification and vitamin E. Hepatology.

[31] Ratziu V., Bellentani S., Cortez-Pinto H. (2010). A position statement on NAFLD/NASH based on the EASL 2009 special conference. J Hepatol.

